# Supplementary material for: Different Responses to Water Deficit of Two Common Winter Wheat Varieties: Physiological and Biochemical Characteristics
Source: Plants (Basel). 2023 Jun 7;12(12):2239. doi: 10.3390/plants12122239 (PMC10304252; doi:10.3390/plants12122239)
Supplement: Supplementary file 1 [file plants-12-02239-s001.zip › plants-2419610-supplementary.pdf]

plants

Supplementary material

Different Responses to Water Deficit of Two Common Winter Wheat Varieties: Physiological and Biochemical Characteristics

Antoaneta V. Popova [1,†], Gergana Mihailova [2,†], Maria Geneva [2], Violeta Peeva [2], Elisaveta Kirova [2], Mariana Sichanova [2], Anelia Dobrikova [1,*], Katya Georgieva [2]

[1] Institute of Biophysics and Biomedical Engineering, Bulgarian Academy of Sciences, Academic Georgi Bonchev Str., Bl. 21, 1113 Sofia, Bulgaria
[2] Institute of Plant Physiology and Genetics, Bulgarian Academy of Sciences, Acad. Georgi Bonchev Str., Bl. 21, 1113 Sofia, Bulgaria
* Correspondence: aneli@bio21.bas.bg (A.D.)
† These authors contributed equally to this work.

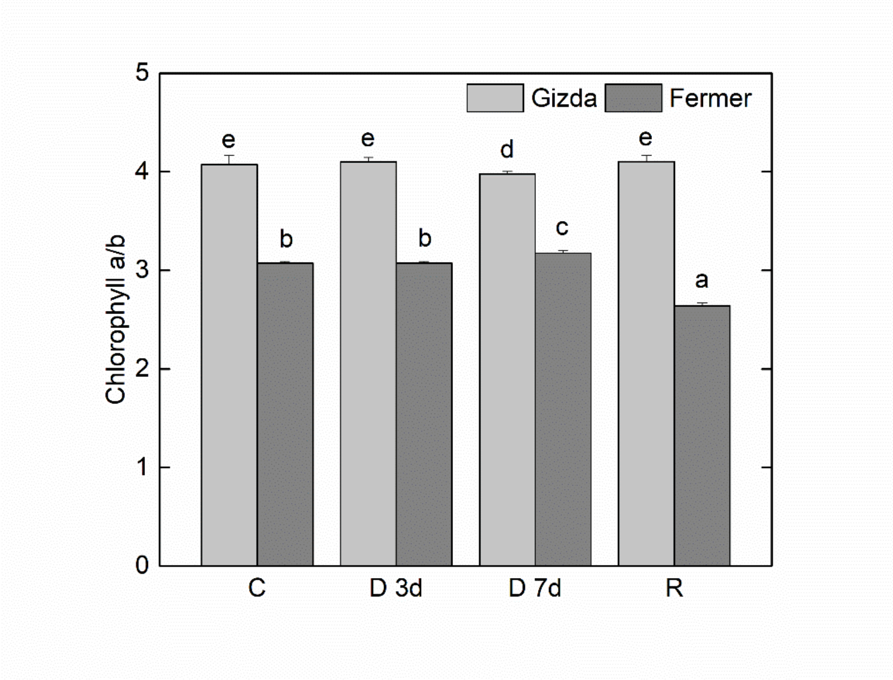

Figure S1. Chlorophyll *a*/*b* ratio in leaves of Gizda and Fermer wheat varieties, subjected to dehydration for 3 (D 3d) and 7 (D 7d) days and after rewatering for 3 days (R). As a control (C) are included values for normally watered plants of the same age. Values are presented as mean ± SE ($n = 4$). Significant differences between values are indicated by different letters according to Fisher's LSD test ($p \leq 0.05$) of multifactor ANOVA analysis.

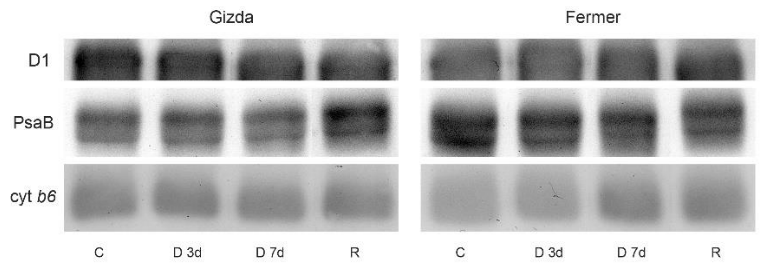

Figure S2. Representative Western blots of the main thylakoid-related proteins D1, PsaB and cyt *b6* of wheat varieties Gizda and Fermer in control (C), dehydrated for 3 days (D 3d) or 7 days (D 7d) and rehydrated (R) plants. Thylakoid samples corresponding to 1.5 μg Chl were applied per lane.
